# Two case reports of chorea-acanthocytosis and review of literature

**DOI:** 10.1186/s40001-022-00646-7

**Published:** 2022-02-07

**Authors:** Shuangfeng Huang, Junliang Zhang, Manli Tao, Yaodong Lv, Luyao Xu, Zhigang Liang

**Affiliations:** 1 Second Clinical Medical College, Binzhou Medical University, Yantai, Shandong China; 2 Department of Neurology, The Affiliated Yantai Yuhuangding Hospital of Qingdao University, Yantai, Shandong China

**Keywords:** Chorea-acanthocytosis, Neurology, *VPS13A*, Gene mutations

## Abstract

**Background:**

Chorea-acanthocytosis (ChAc), as the most common subtype of neuroacanthocytosis syndrome, is characterized by the presence of acanthocytes and neurological symptoms. It is thought to be caused by the VPS13A (vacuolar protein sorting-associated protein 13A) mutations. This article reports two confirmed cases of ChAc and summarizes some suggestive features, which provide direction for the diagnosis and treatment of acanthocytosis in the future.

**Case presentation:**

Here, we present two cases of ChAc diagnosed based on typical clinical symptoms, neuroimaging features, genetic findings of VPS13A, and response to the symptomatic treatment.

**Conclusions:**

Chorea-acanthocytosis is a rare neurodegenerative disease with various early clinical manifestations. The final diagnosis of the ChAc can be established by either genetic analysis or protein expression by Western blotting. Supportive treatments and nursing are helpful to improve the quality of the patient’s life. Nevertheless, it is imperative to investigate the impact of neuroimaging and neuropathological diagnosis in a larger group of ChAc in future studies.

## Background

Chorea-acanthocytosis (ChAc) is a rare genetic disease caused by loss-of-function-mutation of the vacuolar protein sorting-associated protein 13A (*VPS13A*) which is an encoding gene of the protein chorein [1, 2]. The ChAc mainly occurs in adulthood with an average age of about 35 years, and rarely occurs before the age of 20 years or after the age of 50 years [3]. Clinical manifestations of ChAc include progressive chorea, orofacial lingual dyskinesia, seizures, cognitive impairment, psychiatric symptoms, and neuromuscular manifestations with elevated serum biochemical indicators and increased acanthocytes in peripheral blood [1, 4, 5]. Neuroimaging showed atrophy of the caudate nucleus and decreased glucose uptake in the corpus striatum in the basal ganglia of the brain [6].

The diagnosis of ChAc is mainly based on clinical manifestations and laboratory findings of increased acanthocytes in peripheral blood, as well as the exclusion of other diseases that present similar clinical features. In recent years, molecular neuroimaging and genetic testing have played an increasingly important role in the diagnosis of ChAc. The differential diagnosis of ChAc includes McLeod syndrome (MLS), pantothenate kinase-associated neurodegeneration (PKAN), Huntington’s disease-like 2 (HDL2), other forms of inherited chorea (such as Huntington's disease), other forms of Huntington-like disorders, other syndromes of neurodegeneration with brain iron accumulation (NBIAs), Wilson disease, and acquired conditions caused by infection, immunization, drugs, etc. Currently, the treatment of ChAc is limited, mainly drugs and deep brain stimulation (DBS) are used to relieve its symptoms.

The incidence of ChAc is very low and the current knowledge about the disease is mainly based on the small number of case reports. There is no systematic review exploring the pathogenesis, clinical manifestations, imaging characteristics, and management of ChAc in the literature to the best of our knowledge. To improve the understanding of this unique congenital disease, this paper reports two cases of ChAc and discusses its clinical manifestations and the pattern of the genetic cause by analyzing laboratory findings, imaging reports, and relevant literature.

## Case presentation

### Case 1

A 45-year-old female has been suffering from perioral chorea, dysphagia, dysarthria, vocalization, and involuntary upper limb movements during the last 4 months. Her orofacial chorea was characterized by clumsy, non-coordinated mandibular movement, uncontrolled frequent lip biting and occasional grimacing activities. These symptoms worsened during stress and disappeared during sleep. The patient had a history of induced abortion 4 months ago. The patient had frequent oral ulcers when she was overworked, which healed in a few days without any systematic treatment. The subject was born to non-consanguineous parents. The family history did not show similar conditions, and there was no history of drug exposure that would have caused extrapyramidal dysfunction symptoms.

A neurological physical examination showed chorea, head drops, tongue–lip biting, orofaciolingual dyskinesias, slurred speech with vague words, and mild drooling. There were sporadic ulcers on the buccal mucosa and several cracks on the lips. When sitting unsupported, sudden involuntary forward flexions of the trunk were observed. The muscle strength of the extremities was normal, and the muscle tension was reduced. Tendon reflexes were reduced, and sensory response and autonomic nerve function were normal. There was no Kayser–Fleischer (K-F) ring in both corneas. The advanced cognitive function was hardly impaired, and a Mini-Mental State Examination (MMSE) score was 28/30.

**Fig. 1:**
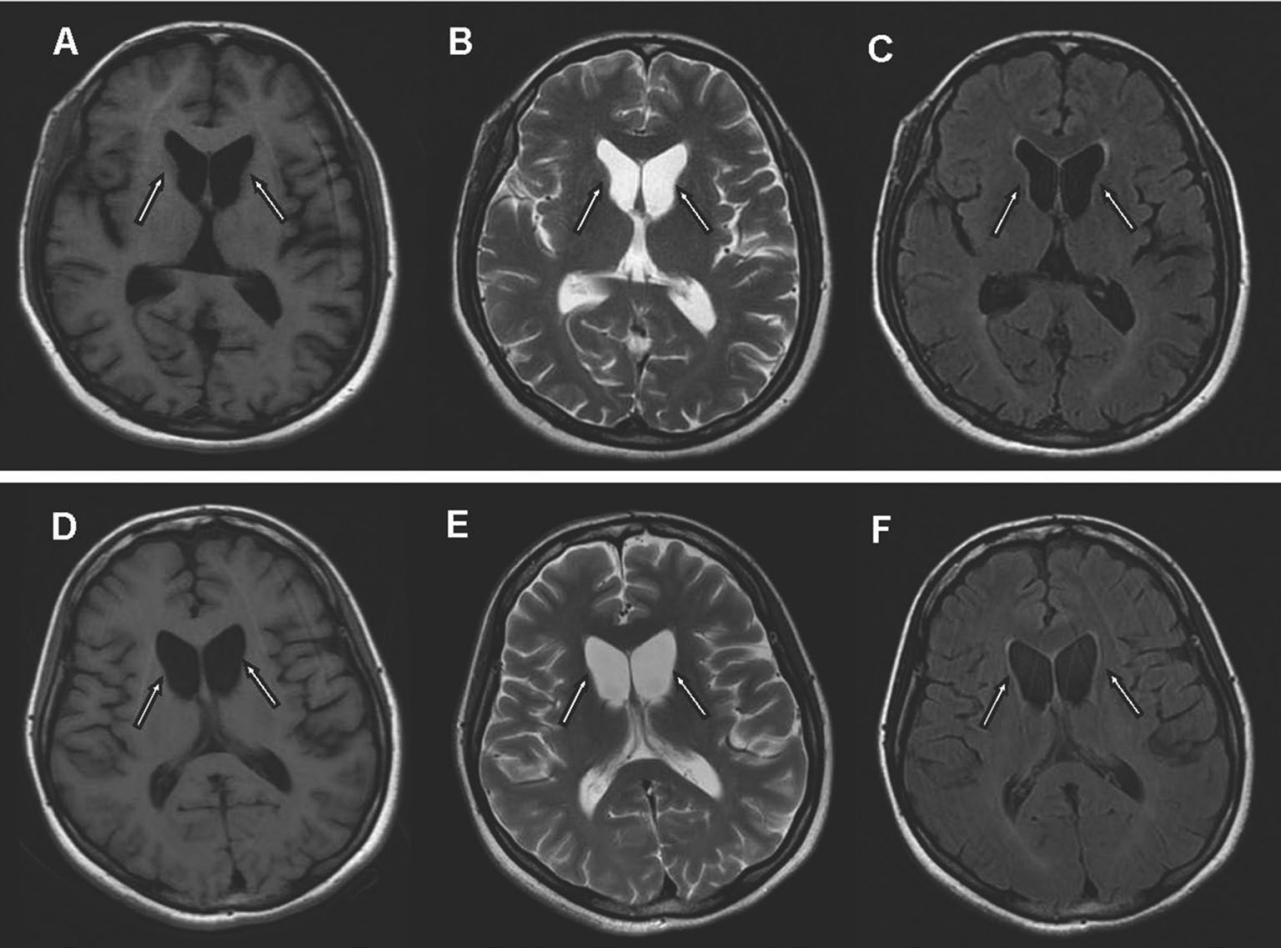
Magnetic resonance images show bilateral atrophy of the head of the caudate nucleus and anterior horn dilation of lateral ventricles (arrows). (**A** and **D**: T1-weighted image, **B** and **E**: T2-weighted image, **C** and **F**: Fluid-attenuated inversion recovery image, **A**, **B** and **C** of patient 1, **D**, **E** and **F** of patient 2)

**Fig. 2:**
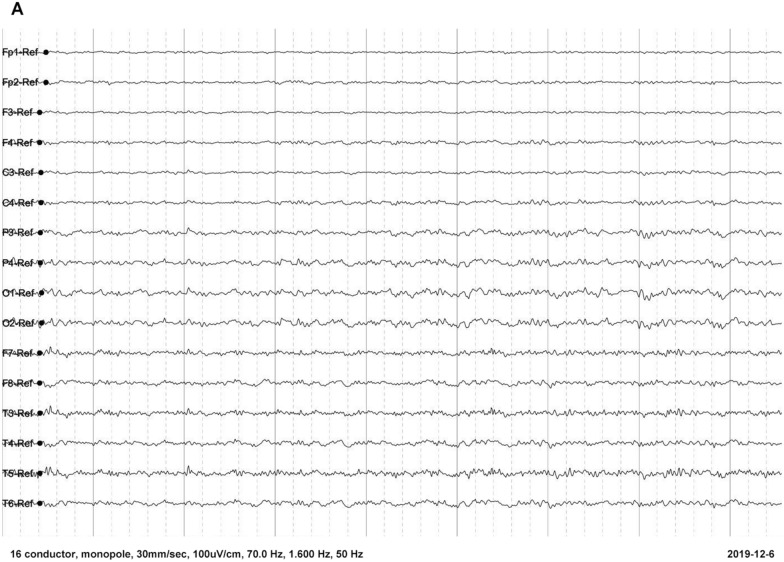
Electroencephalogram of the patient 1

**Fig. 3:**
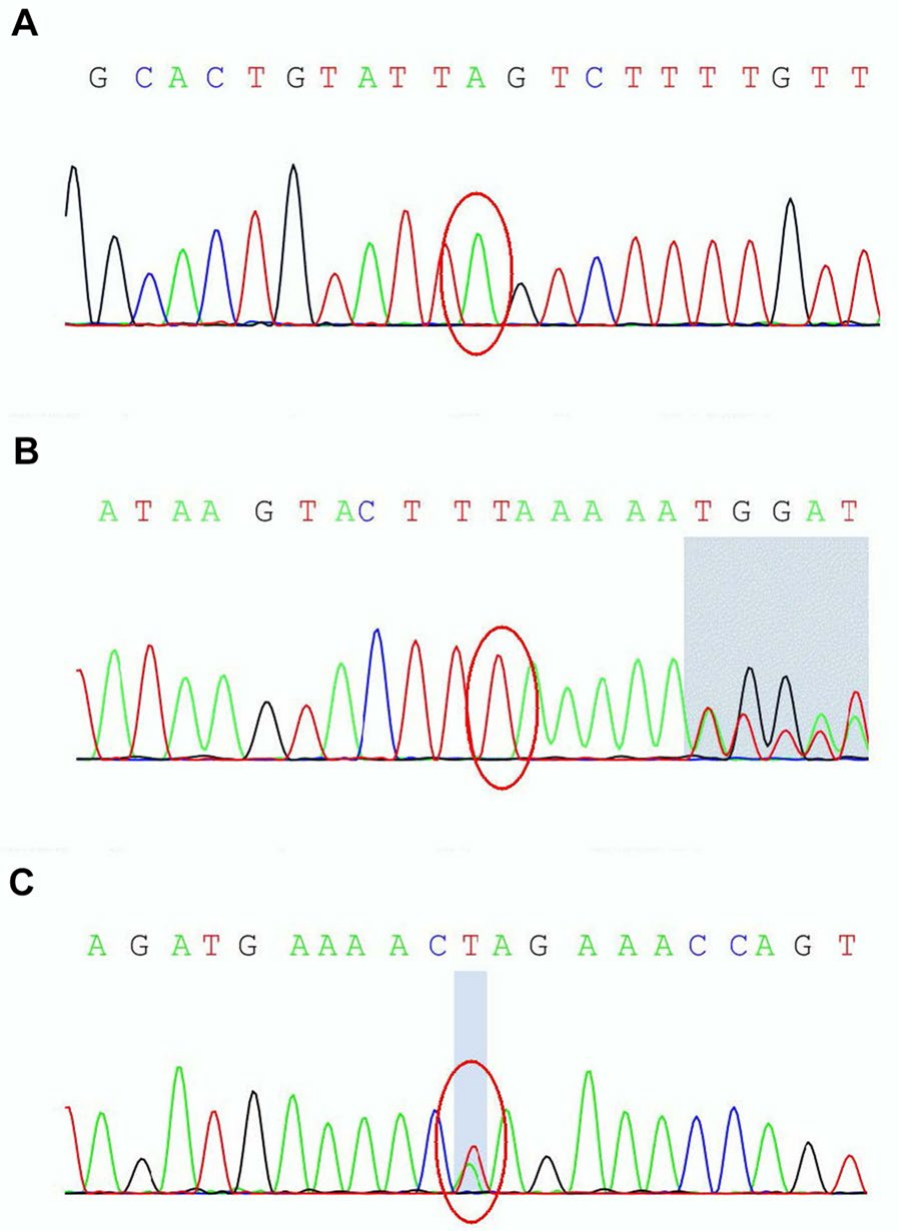
*VPS13A* gene sequence: **A** the genetic test found a hemizygotic mutation (antisense chain) of c. 4063C > T in Chr9:79,922,963 in patient 1. **B** and **C** Two heterozygous mutations were found in patient 2: c. 2833A > T in Chr9:79,895,083 and c.4321_4322insA in Chr9:79,929,484

**Fig. 4:**
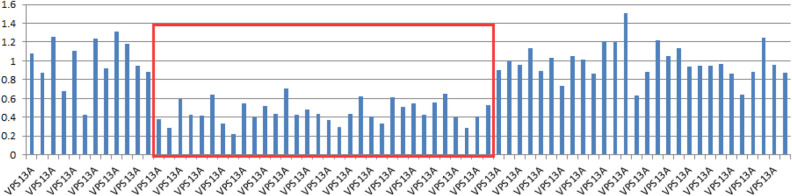
*VPS13A* gene sequence: the genetic test found a heterozygous deletion variant in exons 13–44 of the VPS13A gene in patient 1

On blood, urine, and stool examinations, liver function, renal function, humoral immune function, tumor markers, lupus anticoagulant, erythrocyte sedimentation rate, coagulation function, rheumatoid factor, glycosylated hemoglobin, serum B12 levels, folic acid levels, serum copper, ceruloplasmin, and autoantibody were normal. Electrocardiography, echocardiography, abdominal ultrasonography, and chest radiograph were normal. Abnormal cell findings were as follows: spinous red blood cells accounted for 15.0%; blood biochemistries were as follows: 61.11 g/L total protein (normal: 65–85 g/L), 36.51 g/L albumin (normal: 40–55 g/L), 280 U/L creatine kinase (normal: 40–200 U/L), 917 mg/L lipoprotein (normal: 0–300 mg/L), 10.96 g/L apolipoprotein A (normal: 1–1.6 g/L), and 1.16 g / L apolipoprotein B (normal: 0.6–1.1 g/L). Brain magnetic resonance imaging (MRI) scans showed moderate anterior horn dilation of the lateral ventricles and bilateral atrophy of the caudate nucleus in the brain (Fig. 1A–C). EEG showed mild abnormalities, including occipital area dominated by 9–10 Hz medium and high amplitude alpha rhythm, scattered short-range 18–20 Hz beta activity in each area, and scattered 5–7 Hz medium amplitude theta wave in the forehead (Fig. 2). On genetic testing, a hemizygotic mutation was found in the exon 13–44 of the *VPS13A* gene: c.4063C > T (cytosine > thymine) (Fig. 3A); a heterozygous deletion variant in the exons 13–44 of the *VPS13A* gene (Fig. 4).

Based on the patient’s medical history, clinical manifestations, neurological examinations, cranial MRI, and genetic test, the patient was diagnosed with ChAc. The patient was prescribed idebenone, vitamin B1, vitamin E, mecobalamin, tiapride and clonazepam. Idebenone 30 mg, vitamin B1 10 mg, vitamin E 100 mg and mecobalamin 0.5 mg were given orally 3 times a day for 2 months to relieve the symptoms. Tiapride 100 mg was orally administered 3 times a day until the follow-up time. Clonazepam was started at an initial dose of 0.5 mg taken three times a day. After the first 3 days of therapy, she was treated with clonazepam in doses of 1 mg morning and evening and 0.5 mg in the noon for one month during which time she had no attacks, followed by a maintenance dose of 0.5 mg three times per day until the follow-up time. During the follow-up period, her condition gradually improved. The frequency and amplitude of involuntary movements of limbs were alleviated compared with the initial manifestation. Tongue biting movements and slurred speech dramatically improved than before and no support was required to sit up normally.

### Case 2

The patient was a 33–year-old female who developed drooling after fatigue 3 years ago. As the fatigue disappeared, she still drooled intermittently, and there were no other accompanying symptoms. Two years ago, she began to show involuntary movements including tongue and limbs, writing jitter and clumsy fine movements. Dysphagia and lower limb weakness occurred 2 months ago, however, which did not affect her daily life. It was self-reported that taking tiapride, clonazepam, sertraline did not improve the symptoms. Take tiapride 100 mg, clonazepam 0.5 mg and sertraline 50 mg every day for 10 days. The patient was not exposed to drugs causing extrapyramidal dysfunction symptoms. A further family history inquiry revealed no similar symptom presenters.

A neurological examination showed systemic chorea characterized by involuntary movement of limbs and tongue. There were sporadic ulcers on the buccal mucosa and several cracks on the lips. The muscle strength of the extremities was normal, and the muscle tension was reduced. Tendon reflexes were not elicited. Coordination function, sensory response, and autonomic nerve function were all normal. There was no K-F ring in both corneas of the eyes. The advanced cognitive function was almost unimpaired, and an MMSE score was 27/30.

**Fig. 5:**
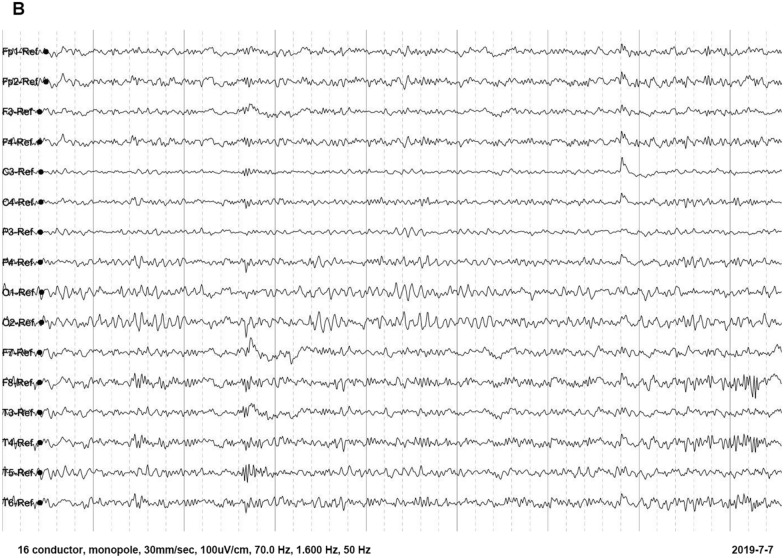
Electroencephalogram of the patient 2

There was no abnormal cell finding. Biochemical findings: 488 µmol/L uric acid (normal: 149–368 µmol/L), 1015 U/L creatine kinase (normal: 40–200 U/L), and 283 U/L lactate dehydrogenase (normal: 120–250 U/L). MRI of the brain showed moderate anterior horn dilation of the lateral ventricles and bilateral atrophy of the caudate nucleus (Fig. 1D–F). EEG showed much more long-range and short-range 5–7 Hz theta waves with medium amplitude in each area, mixed with a small amount of 16–20 beta waves with low amplitude (Fig. 5). Genetic testing: two heterozygous mutations were found in the exon region of the *VPS13A*: c.4321_4322insA and C.2833A > T (adenine > thymine) (Fig. 3B, C). The results of the remaining auxiliary examination items were similar to those of the previous subject and were normal.

Based on the patient’s medical history, clinical manifestations, neurological examination, cranial MRI findings, and genetic testing results, the diagnosis was confirmed as ChAc. The patient was prescribed clonazepam, mecobalamin, and vitamin B1. She received oral administration of mecobalamin (0.5 mg, 3 times/day) and vitamin B1 (10 mg, 3 times/day) for 2 months. Clonazepam was orally administered at an initial dose of 0.5 mg taken three times a day. After the first 3 days of therapy, she was treated with clonazepam in doses of 1 mg morning and evening and 0.5 mg in the noon for one month during which time she had no attacks, followed by a maintenance dose of 0.5 mg three times per day until the follow-up time. After a year and a half of follow-up, she was aware of her condition that gradually improved, especially the symptoms of drooling and involuntary shaking.

## Discussion and conclusions

In this study, we present two cases of ChAc diagnosed based on clinical symptoms, neuroimaging features, genetic findings of *VPS13A*, and response to the symptomatic treatment.

### Genetic features

The ChAc is a rare autosomal recessive inherited neurological disorder [7] caused by *VPS13A* mutation [3, 8]. The total length of the gene is about 250 kb, which is composed of 73 exons [8–10]. *VPS13A* is precisely located in two laboratories [2, 11], and the encoded protein is named "chorein". The types of *VPS13A* mutations include missense, nonsense, frameshift, splice site, replication, and deletion mutations [10, 12]. These mutations have been reported to eliminate or severely alter chorein expression [13]. The chorein is expressed in several tissues [14], especially in the brain tissue and red blood cells, but the expression is absent or severely down-regulated in the tissues of ChAc patients [13]. The absence of chorein or significant reduction of chorein protein level can be shown using Western blots [13]. The DNA analysis of the *VPS13A* gene is difficult due to the size of the *VPS13A* gene and the heterogeneity of mutation sites [15], and therefore, chorein protein detection may be the only promising option to detect the altered gene expression. The morphology of erythrocytes is maintained by membrane lipids, proteins, and spectrin–actin cytoskeleton. Chorein protein involves in the intracellular transport by many transmembrane proteins and affects actin polymerization [16]. Further, VPS13A participates in regulating the level of PtdIns phosphorylation on the plasma membrane of the erythrocytes [17]. The level of PtdIns [4]P regulates the interaction between the plasma membrane and cytoskeleton, and the altered expression of VPS13A results in the destruction of the cell membrane and abnormal morphology of red blood cells [18].

These patients presented in this report were two sporadic cases with no family history of this neurological condition and likely had an autosomal recessive inheritance pattern. In this study, a heterozygous mutation, and a heterozygous deletion variant in exons 13–44 of the *VPS13A* gene were found in patient 1 (c.4063C > T), while two heterozygous mutations in the exon region were found in patient 2 (c.4321-4322insA and c.2833A > T). In general, the mutations in these patients may probably give rise to premature translation termination, nonsense-mediated mRNA decay (NMD) pathway induced *VPS13A*-mRNA degradation [19], abnormal spliceosome formation, and production of aberrant chorein, which can genetically contribute to the pathogenesis of ChAc. The deletion of exon 13–44 of the *VPS13A* gene has not been reported. If the mutation c.4063c > T and deletion mutation are pathogenic, it will theoretically explain the pathogenesis of ChAc in patient 1. Since patient 1 had a history of induced abortion before the onset of the disease, we can conclude that the conditions such as exertion, agitation, stringent state (e.g., childbirth and miscarriage), and malnutrition can accelerate the progression. Parents did not have a genetic test because of the expenses and the absence of the genetic result is the limitation of this study.

### Clinical features

The clinical manifestations of ChAc are diverse, and there is no common diagnostic standard. Progression of clinical symptoms such as chorea, orofaciolingual dyskinesias (involuntary movements of eyes, mouth, tongue including vocalization), limb or facial dystonia is the main clinical characteristic [15]. Perioral chorea, head drops, and writing jitter were the concrete manifestations of the hyperkinetic movements in both patients. Several reports have shown that tongue and lip biting is suggestive indicators of ChAc [3, 20]. Patients can develop Parkinsonism in the late stage, which may be related with involvement of nigrostriatal pathways as disease progresses.

Seizure, neuropathy, psychiatric symptoms and dilated cardiomyopathy are also relatively common manifestations of ChAc [15]. In some patients, the disorder begins with the above atypical symptoms, and it challenges making a definitive early diagnosis [21]. It has been speculated that about 42% of patients have at least one seizure attack in the middle stage or the late stage of the disease [1]. Most of the seizures are antiepileptic drugs (AEDs)-sensitive generalized tonic–clonic seizures (GTCS), which mainly result from the abnormal discharges in the temporal lobe [22]. Connolly et al. [23]found that there was a significant loss of parvalbumin-positive intermediate neurons in the cerebral cortex and striatum in the ChAc patients experiencing epilepsy. It is also likely that patients may experience other peripheral neuropathies, such as distal amyotrophy and progressive loss or absence of reflexes. Psychiatric symptoms are mainly characterized by emotional instability, apathy, anxiety, depression, and mania. About half of the patients have mild-to-moderate mental disorders. Remarkably, psychiatric manifestations often precede movement disorders, leading to high risk of misdiagnosis sans thorough examination and continuous follow-up. The cognitive function of both patients evaluated by the MMSE scale was still normal, and the patients may have been in the early clinical course.

### Laboratory workups and neuroimaging

Several laboratory workups can assist the diagnosis of ChAc. Elevated serum creatine kinase can be seen in most patients with ChAc, which may be used as a sign of subclinical myopathy before the onset of clinical neurological symptoms [15, 24]. Creatine kinase was elevated in both patients in this study. Acanthocyte detection in peripheral blood smear has been shown to be helpful for diagnosis of ChAc, while it is very insensitive (positive rates as low as 5%–50%) and acanthocytes may only appear late in the disease course [25] and may occasionally be detected in other diseases, such as mitochondrial diseases and metabolic disorders [26]. The detection efficiency of acanthocytes is largely dependent on several specific endogenous factors and blood sample treatment methods [27], and their absence does not absolutely exclude the diagnosis of ChAc as there are also cases where no acanthocytes may be detected [15, 25]. A close monitoring is suggested when suspecting ChAc. The scanning electron microscope is the most reliable tool for finding morphological features of acanthocytes, but it is not frequently used at present. We have successfully identified acanthocytes in peripheral blood smears in patient 1.

In neuroimaging, MRI of the brain showed atrophy of heads of caudate nuclei and dilation of the anterior horn of the lateral ventricles [28]. Neuropathological examination showed neuron loss and glial hyperplasia in the putamen, globus pallidus, and substantia nigra, and these changes were more obvious in the caudate nucleus [29]. Cortical atrophy has also been reported, but to a much lower degree [3], and iron deposits in the striatum and globus pallidus, have occasionally been reported [29]. FDG-PET/CT also demonstrates impaired glucose metabolism in the basal ganglia. Such results indicate that these advanced MRI techniques could be promising diagnostic indicators. The MRI of both patients showed dilated anterior horn of the lateral ventricle and atrophy of the caudate.

### Differentials diagnosis

To make an accurate diagnosis, either VPS13A mutation confirmed by gene sequencing, or chorein deletion in red blood cells proven by Western blotting is required. However, the detection methods at gene/protein level have not been popularized. Most diagnosis are made according to typical clinical manifestations, peripheral blood cells and radiological features, so we make differential diagnosis from this point of view.

Huntington disease (HD) is a classic differential diagnosis of ChAc, which is an autosomal dominant inherited disorder associated with gene IT15 mutation. HD has a common onset age of 35 to 45 years. Epileptic seizure and peripheral nerve impairments are rarely seen. Myopathy is not common and serum creatine kinase level is usually normal. Chorea symptoms of the limbs are more prominent in HD patients. Peripheral acanthocyte level is normal in most cases. Brain pathology shows a generalized brain atrophy with predominance in the basal ganglia, in particular the tail and body of the caudate rather than the head of the caudate nucleus [30]. ChAc patients were characterized by chorea, orofaciolingual dyskinesias (involuntary movements of eyes, mouth, tongue including vocalization) should be different from Wilson's disease. There is no "K-F ring" in cornea, the liver function is normal, and the content of ceruloplasmin is normal, which can be excluded. Major representatives of neuroacanthocytosis syndromes with vastly overlapping clinical features are the autosomal recessive ChAc and the X-linked MLS. MLS is a rare disease of the nervous system, which is X-linked recessive associated with gene XK. Most are male patients aged 25–60 years. Patients with McLeod syndrome have a distinct hematologic presentation with specific transfusion requirements. The symptoms of central nervous system and radiological features of ChAc and McLeod syndrome are not easy to distinguish. The clinical course of MLS patients is longer, and the prevalence of arrhythmia and dilated cardiomyopathy is higher. The two patients in this study were middle-aged women without cardiac manifestations [31].

### Case management

Currently, there is no effective primary treatment for the ChAc and symptomatic supportive treatments are adopted. Involuntary movement can be treated with dopamine antagonists or consumptive agents such as thiacloprid and clozapine. L-Dopa can alleviate dystonia [32], and focal botulinum toxin injection is one treatment modality for the typical orofacial dystonia of ChAc patients [33]. The use of surgical interventions in ChAc has made substantial progress in recent decades. Deep brain stimulation is gradually adopted as a potential treatment, many of which have reported globus pallidus internus (GPi) as the surgical target. The DBS is more effective for patients with drug-refractory involuntary movement [34]. Epilepsy can be treated with valproate and levetiracetam [35]. The treatment of epilepsy in patients with chuck is a challenge because the symptoms of epilepsy in these patients are difficult to control. Besides, some antiepileptic drugs can aggravate chorea. A large dose of vitamin E can change the fluidity of the erythrocyte membrane and lessen the symptoms. It is also necessary to manage patients' self-mutilation behaviors, such as pulling out teeth or biting sticks, towels, and other items. According to relevant literature reports (36), the course of ChAc is 12–20 years, and the longest survival period is 40 years. Generally, the prognosis of this disease is poor, and most of the patients died of aspiration pneumonia and severe malnutrition.

In conclusion, chorea-acanthocytosis is a rare neurodegenerative disease with various early clinical manifestations. When the triad of orofacial dyskinesia, epileptic seizures, and elevated creatine kinase, neuro-physicians should be alerted to the diagnosis of ChAc. The final diagnosis of the ChAc can be established by either genetic analysis or protein expression by Western blotting. Supportive treatments and nursing are helpful to improve the quality of the patient’s life. Nevertheless, it is imperative to investigate the impact of neuroimaging and neuropathological diagnosis in a larger group of ChAc in future studies.

## Data Availability

The datasets for this article are not publicly available due to concerns regarding participant/patient anonymity. Requests to access the datasets should be directed to the corresponding author.

## Abbreviations

ESR: Erythrocyte sedimentation rate
EEG: Electrocardiograph
MRI: Magnetic resonance imaging
GTCS: Generalized tonic–clonic seizure
AEDs: Antiepileptic drugs
MMSE: Mini-Mental State Examination
K-F: Kayser–Fleischer
HD: Huntington disease
